# Supplementary material for: Integrative analysis of microbial 16S gene and shotgun metagenomic sequencing data improves statistical efficiency
Source: Res Sq. 2023 Oct 3:rs.3.rs-3376801. Preprint. [Version 1] doi: 10.21203/rs.3.rs-3376801/v1 (PMC10602108; doi:10.21203/rs.3.rs-3376801/v1)
Supplement: Supplement 1 [file NIHPPRS3376801V1-supplement-1.pdf]

Supplementary Files

This is a list of supplementary files associated with this preprint. Click to download.

- 2023092216Sshotgunsup.pdf
